# Quantifying information transfer by protein domains: Analysis of the Fyn SH2 domain structure

**DOI:** 10.1186/1472-6807-8-43

**Published:** 2008-10-08

**Authors:** Tom Lenaerts, Jesper Ferkinghoff-Borg, Francois Stricher, Luis Serrano, Joost WH Schymkowitz, Frederic Rousseau

**Affiliations:** 1 SWITCH laboratory, VIB, Brussels, Belgium; 2 Vrije Universiteit Brussel, Brussels, Belgium; 3 Ørsted. DTU, Technical University of Denmark, Kgs. Lyngby, Denmark; 4 Systems Biology Program, Centre for Genomic Regulation (CRG), Barcelona, Spain

## Abstract

**Background:**

Efficient communication between distant sites within a protein is essential for cooperative biological response. Although often associated with large allosteric movements, more subtle changes in protein dynamics can also induce long-range correlations. However, an appropriate formalism that directly relates protein structural dynamics to information exchange between functional sites is still lacking.

**Results:**

Here we introduce a method to analyze protein dynamics within the framework of information theory and show that signal transduction within proteins can be considered as a particular instance of communication over a noisy channel. In particular, we analyze the conformational correlations between protein residues and apply the concept of mutual information to quantify information exchange. Mapping out changes of mutual information on the protein structure then allows visualizing how distal communication is achieved. We illustrate the approach by analyzing information transfer by the SH2 domain of Fyn tyrosine kinase, obtained from Monte Carlo dynamics simulations. Our analysis reveals that the Fyn SH2 domain forms a noisy communication channel that couples residues located in the phosphopeptide and specificity binding sites and a number of residues at the other side of the domain near the linkers that connect the SH2 domain to the SH3 and kinase domains. We find that for this particular domain, communication is affected by a series of contiguous residues that connect distal sites by crossing the core of the SH2 domain.

**Conclusion:**

As a result, our method provides a means to directly map the exchange of biological information on the structure of protein domains, making it clear how binding triggers conformational changes in the protein structure. As such it provides a structural road, next to the existing attempts at sequence level, to predict long-range interactions within protein structures.

## Background

Cooperative protein response and thus cooperative network behavior requires information transfer between distal sites in a protein or protein complex. Protein structures often achieve such long range communication by allosteric movement [1-4], but this is certainly not a requirement. Essentially any change in the dynamic properties of protein residues, upon ligand binding for example, that efficiently propagates through the structure and is detectable at a distal site, constitutes a form of signal transduction [5-9]. Many theoretical and experimental studies have been devoted to the structural dynamics of globular proteins and its implications for protein function. Recent experimental studies, on the one hand, focus on the role of protein dynamics for catalysis [10], signal transduction [7,11], cooperative response [12] and protein aggregation [13]. On the other hand, theoretical approaches have probed the relationship between protein structural dynamics and signal transduction, limited only by the combinatorial complexity associated with the conformational space. In response, techniques such as targeted molecular dynamics [11], anisotropic thermal diffusion [5] or Go-like sampling [14] were developed to reduce the degrees of freedom so as to allow the mapping of a pathway that connects one ground state to its allosteric counterpart. Additionally, Ranganathan and co-workers have mapped residues that participate in signal transduction in several important proteins by extracting evolutionary correlated mutations from multiple sequence alignments [7]. Together these studies show that (1) changes in protein dynamics can propagate through the protein structure thereby creating long range correlations between distal active sites [6,14], (2) only a fraction of residues in a protein structure participate in signal propagation [9,12], and (3) these intra-protein communication modes are generally conserved within protein families and even protein folds [4,7].

Now that the principles relating correlated structural dynamics to signal transduction mechanisms within proteins are becoming apparent, we here present a method to identify and quantify these structural fluctuations in terms of information allowing computation of the information transfer between active sites. In particular, we use information theory [15], and more specifically the concept of mutual information, as a means to describe the relationship between structural protein dynamics and signaling behavior. This association directly follows from the relation between the definitions of entropy as a measure of structural disorder in statistical thermodynamics, on the one hand, and as a measure of error on communication channels in information theory on the other hand [16]. The potential of globular proteins to convey information throughout their structure, thereby correlating the behavior of distant effector sites, is indeed provided by the change in structural dynamics induced by ligand binding. But more fundamentally, information transfer originates from mutual conformational dependence of the different residues composing a protein or protein complex. In other words, when the conformational flexibility of one residue affects the conformational flexibility of another residue, changes in the structural dynamics will produce correlated changes in entropy and information is thus exchanged [17]. Mutual information quantifies the amount of conformational dependence between protein residues. As a result a protein can be considered as a network of residues exchanging conformational information. This interpretation provides a theoretical framework to understand how the redistribution of thermodynamic perturbations within a network of protein residues constitutes an information transfer network.

**Figure 1:**
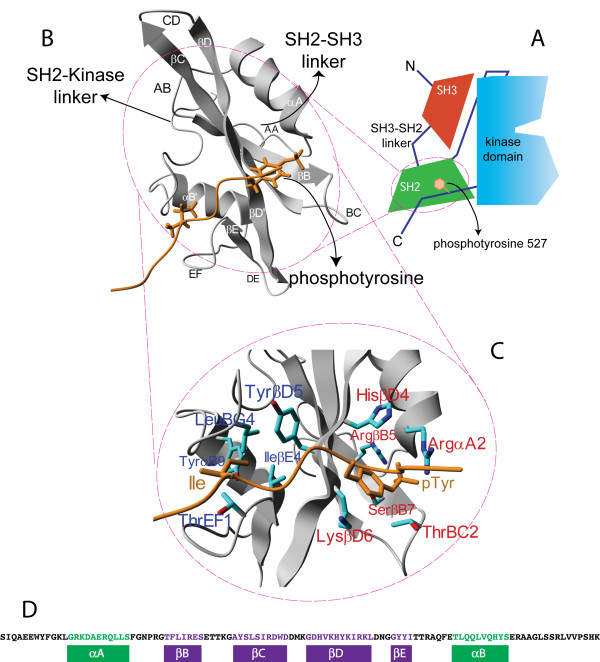
**The Fyn tyrosine kinase and its SH2 domain**. The Fyn tyrosine kinase **(A) **consists of three domains: the N-terminal domain SH3, an SH2 domain that binds phosphotyrosine motifs, the SH1 domain with tyrosine kinase activity and a short C-terminal tail. When inactive, the tyrosine at position 527 in the C-terminal part is phosphorylated and bound to the SH2 domain. In **(B) **the SH2 domain together with the bound C-terminal phosphotyrosine peptide is visualized. The standard nomenclature for the secondary structure elements is added, see also **(D)**. **(C) **Close-up view of the phosphopeptide and hydrophobic binding sites, including the sidechains of the residues relevant for binding. The standard nomenclature of the key interacting residues is provided in [25] (PDB identifier 1AOT [38]). All molecular graphics created with YASARA  and PovRay .

To illustrate our information theoretic approach, we map the residue-based information transfer network of the SH2 domain of the Fyn tyrosine kinase [18], a member of the Src family of kinases [19], that are involved in essential signaling pathways, for example controlling cell growth, proliferation, differentiation, motility and cell adhesion [20]. Family members share a common multi-domain architecture, with the archetypal Src homology 3 (SH3) domain at the N-terminal [21], a Src homology 2 (SH2) domain [22,23], the tyrosine kinase domain and a short C-terminal tail. In its inactive form the kinase domain is sequestered by the SH3 and SH2 domains (see Figure 1A). The SH3 domain docks to the linker connecting the SH2 to the kinase domain. The SH2 domain holds on to a C-terminal phosphotyrosine sequence (see Figure 1A). Dephosphorylation of this C-terminal sequence leads to its release from the SH2 phosphopeptide-binding site. This in turn leads to the release of the SH2-kinase linker from the SH3 domain and undocking of the kinase domain which is more than 40 Angstrom (see Figure 1A) away from the SH2 phosphopeptide binding site [24].

**Figure 2:**
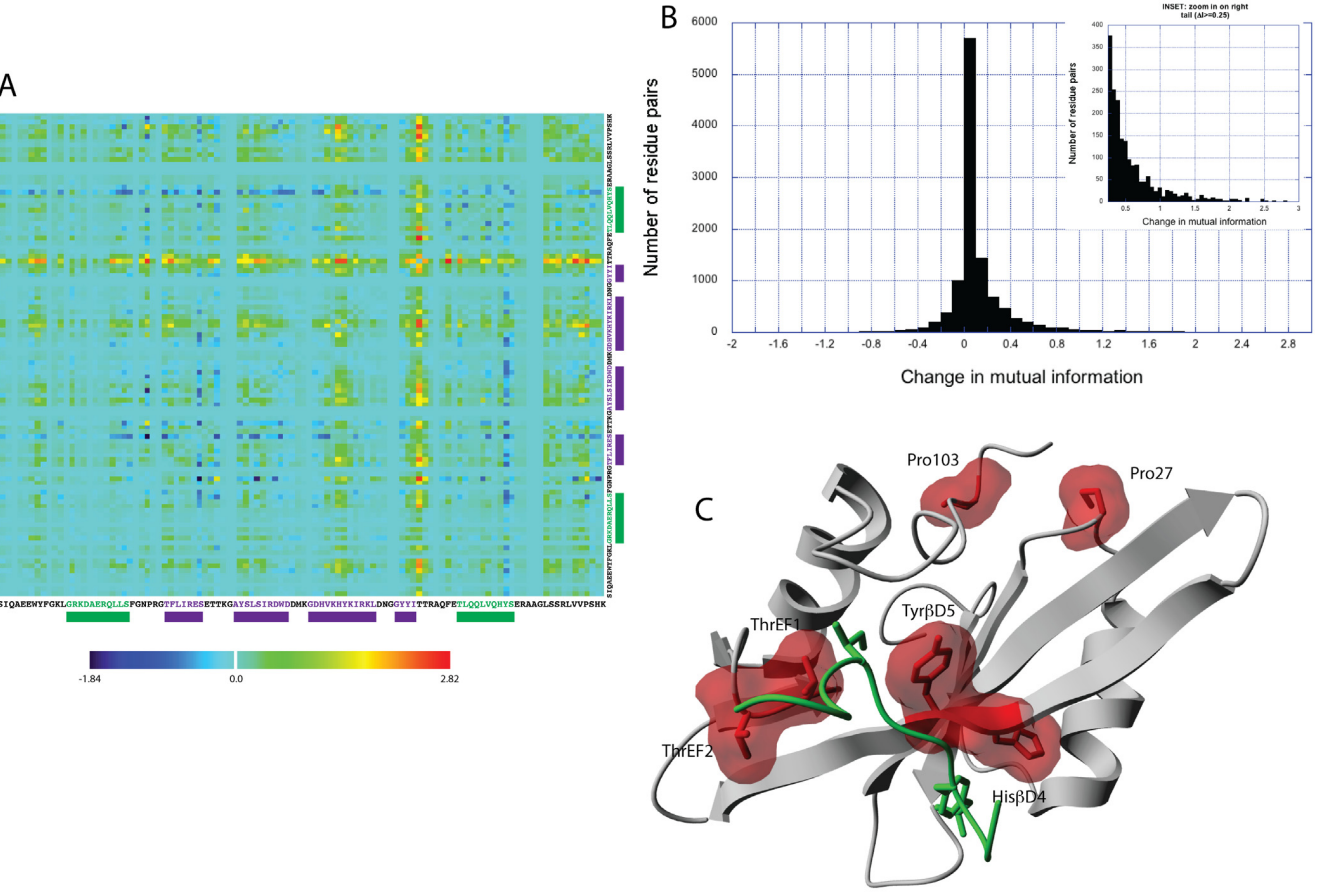
**Change in mutual information per residue and most significant cluster**. Binding the peptide with phosphorylated tyrosine to the SH2 domain results in a change in mutual information residue-residue interactions. **(A) **This panel shows the change in mutual information for each pairwise interaction. Clearly most residues remain unaffected. A number of residues at particular positions experience the strongest effect. **(B) **Most residues experience almost no change in mutual information. The inset shows the distribution in the tail starting with a change in mutual information bigger than 0.5 (noise level) **(C) **Clustering the residues using the change in mutual information as similarity measure (see Methods), results in a cluster of highly coupled residues, located in the binding pockets and near the kinase-linker region of the SH2 domain. All molecular graphics created with YASARA  and PovRay .

The SH2 domain has a typical length of approximately 100 amino acids and displays a simple fold (shown in Figure 1B) in which a central anti-parallel β-sheet is flanked by two alpha-helices. The β-strands are commonly annotated from βA to βG, whereas the α-helices are referred to as αA and αB. Panel B (and D) of Figure 1 shows the labeled structure (sequence) of the Fyn SH2 domain that is used in the present analysis (see Methods for structure information). The peptide binding site of the Fyn SH2 domain consists of two functional regions: the phosphotyrosine binding pocket (red labels in Figure 1C), responsible for pTyr recognition, and the hydrophobic binding pocket, which interacts with the residues C-terminal to the pTyr (blue labels in Figure 1C) [25,26]. The first binding cavity is located between the central anti-parallel β-sheet and the α-helix αA. The second binding pocket is located on the opposite side of the phosphotyrosine binding cavity, between the central β-sheet and the α-helix αB. In Figure 2C, the names of the residues involved in binding the C-terminal phosphorylated sequences are annotated onto the structure following the nomenclature defined in [25,26]. These names, e.g. HisβD4, will be used when referring to residues of either binding cavity. Other residues, e.g. Ser23, will be indicated by their amino acid name and position in the Fyn SH2 PDB structures used here (see Methods). For further detailed discussion of SH2 function and nomenclature we refer to the literature [25,26].

Experimental work on the role of the linker between the SH3 and SH2 domain of Fyn has shown that the nature of the linker, i.e. the residue pattern, determines both orientation and coupling between the two domains [27,28]. As such it has a direct effect on the activation behavior of the protein kinase and on the intra-molecular communication. This observation is supported by experimental and molecular dynamics work performed by Kuriyan et al. [9] on the SRC kinase. They showed that the release of the C-terminal sequence from the SH2 binding site uncouples the motions of the SH2 and SH3 domains by increasing the flexibility of the linker loop connecting these two domains. The connector link between the SH2 and SH3 domain therefore acts as an inducible snap lock mechanism and replacement of three of the eight residues in this linker by glycines generates a constitutively active kinase. In effect, the phosphopeptide binding site at one end of the SH2 domain is thus coupled to the SH3-SH2 linker at the opposite side of the SH2 domain. Importantly, cooperativity is achieved without major allosteric movement within the SH2 domain and therefore originates from subtle differences in dynamics between phosphopeptide bound and unbound states. Our analysis shows that mutual information identifies and quantifies the cooperativity in the SH2 domain over long distances, confirming other results for different non-allosteric structures [29,30]. In other words, binding the phosphotyrosine peptide triggers an information exchange from the SH2 binding pockets toward residues located at the opposite side of the domain, targeting the linker regions. Hence, the SH2 peptide binding site acts as a switch that distributes information on its binding state to both linkers with the other domains, coordinating the SH2-SH3 docking module.

## Results and Discussion

### How to quantify the information exchange within a protein?

In Shannon's theory of information transduction along noisy channels, where the level of noise corresponds to the fraction of the input characters that may be wrongly translated to output characters, information is quantified as the degree of certainty obtainable about the output signal, once a particular input is given [15,17]. A random channel that produces for any input signal a random output has a maximum degree of uncertainty, whereas for a noiseless channel, which produces an exact copy of the input at the output, there is no uncertainty about the output. This variation in the degree of uncertainty is expressed in terms of entropy, allowing for the immediate application of information theoretical principles to protein dynamics simulations in a straightforward manner [16]. Specifically, we focus here on the notion of mutual information to express the mutual dependence between the conformational states of residues in a protein, as a quantification of the information flow within. For more information on information theory we refer to [17]. As a tool, mutual information, and other co-variation measures, has been applied to identify co-varying or co-evolving residues in multi-sequence alignments [31,32]. Yet, as far as we are aware, no work exists that applied this informational theoretical measure to determine the conformation coupling between residue sidechains.

So when do two sidechains exchange information? Two linked residues are dependent when knowing one conformation will convey information about the conformation of the other. In this case, the two residues share information. For instance, consider two amino acid residues in a protein, each having some conformational variation in their side chains. The mutual information shared by these residues is defined by the entropy reduction that is observed at the second residue when the conformation of the first is fixed in an arbitrary configuration (and vice versa). Thus, when ligand binding conformationally restricts the first residue then mutual information will quantify how much of the signal of ligand binding is received at the second residue since its conformational flexibility will also be restricted. Mutual information between two residues is thus zero when the conformational state of one residue does not provide any information about the conformational state of the other residue. On the other hand, mutual information is maximal when each conformational state of a residue's sidechain uniquely defines the conformational state of the other residue's sidechain. In accordance with information theory, the conformational space of each residue corresponds to the residue's alphabet.

### Exploration of the conformational space of the amino acids

The calculation of mutual information between any two amino acids in a protein requires reliable statistics of the conformational dynamics of all amino acids in the native structure of the protein (see Methods for details). The method relies on computing both the probability of residue i to adopt an arbitrary conformation independently and the joint probability of finding residue i and residue j in an arbitray combined combination. This is achieved via effectively sampling the conformational states of each amino acid in a protein structure so that all possible combinations of conformations of residues i and j can be explored. It is clear that the computational size of such a sampling problem can only be addressed by reducing the number of possible conformations that each amino acid can adopt to a relatively small number of discrete states. To achieve this, we treat the sidechain and backbone flexibility of each amino acid as separate components of its overall conformational dynamics and consider only a finite number of states for each.

In order to keep the number of sidechain conformations computationally tractable, it is common to employ a discrete, finite-size alphabet for each residue, called rotamer library. Such rotamer libraries are constructed by extracting from a database of high resolution protein structures the most frequently occurring states and thus the degree of coarse graining that is employed to record the statistics imposes a resolution on the data. For the purpose of detecting conformational dependencies between neighboring residues in a protein structure, we require a finer resolution than is provided by common rotamer libraries used for homology modeling and we thus constructed a database of backbone dependent sidechain conformations with a 10 degree resolution on the sidechain dihedral (Chi-) angles (see Methods). Given the backbone dihedral angles of a certain residue in the protein structure, a list of possible sidechain conformations and their probabilities can be retrieved from this database. The number of chi angles in the residue determines the size of this list, meaning that residues with small sidechains tend to have shorter lists than residues with long sidechains. The conformational state of the sidechain in the protein structure serves as a starting point for generating these lists.

Information concerning the flexibility of the backbone of a protein can be obtained from either molecular dynamics simulations (MD) or NMR data. Exploration of the conformational space accessible to the protein backbone using MD is a computationally expensive method. Moreover, Vendruscolo and co-workers have recently shown that MD simulations yield more realistic results when restricted by experimental data in the form of residue-residue distances derived from Nuclear Overhauser Effects (NOEs) in a Nuclear Magnetic Resonance (NMR) experiment [33,34], suggesting that unrestricted MD simulations are not the most effective method to sample the backbone ensemble. The work of Vendrusculo and others also revealed that the backbone dynamics can essentially be captured by a small number of representative backbone structures. Moreover, Bahar and colleagues recently showed [35] that NMR models can also be viewed as an ensemble of conformations accessible under physiological conditions and that even though the RMSD values reflect the uncertainties in the coordinates, they also contain physically meaningful contributions of equilibrium fluctuations [35]. Although this point is still under debate, we here found that sampling the sidechain conformations of each amino acid on the collection of backbones present in the NMR dataset using a Monte Carlo approach (see Methods) yields results that are consistent with experimental studies as well as more exhaustive simulations performed by Kuriyan et al [9]. More specifically, we employed a Metropolis algorithm implemented in the FoldX force field [36,37] from which an equilibrium distribution of sidechain conformations, compatible with a given protein backbone structure, is obtained (see Methods for details). The force field scores the conformations taking into account the packing interactions.

The sidechain sampling results for a single backbone structure obviously introduce a strong bias in the apparent information flux towards residues whose sidechains arbitrarily happen to be strongly coupled in this particular backbone conformation. When information is obtained from an ensemble of related backbone conformations however, each backbone introduces slight variations in the pattern of residue-residue couplings. As a result, the combination of the sidechain sampling on the entire ensemble of backbone structures acts as a filter that removes sporadic couplings while accumulating consistent couplings, thereby revealing the true network of information exchange between all residues. We have here taken the entire ensemble of backbone structures in the NMR datasets of the Fyn kinase SH2 domain as an adequate sample and have not systematically explored if a reduced number of backbones could be employed to the same effect.

### Construction of the residue-residue information network in Fyn SH2

Since the Monte Carlo sampling directly yields the equilibrium distribution of sidechain conformations observed at each position along the protein backbone, the quantity of mutual information between all residue pairs in the protein can be calculated using probability and entropy calculations (see Methods and [17]). In this way, information transfer over both short and long distances in the folded protein is elucidated, explicitly revealing the communication between all residues in the protein. Extracting only the changes in mutual information that result from the difference between bound and unbound states of the SH2 domain (see Methods), highlights the change in communication patterns. Hence, those amino acids that experience the strongest changes in mutual information can be detected and mapped on the network of sidechain interactions in the protein, thereby revealing how series of dynamically coupled residues in the topology of the protein direct the overall communication.

In order to obtain the network of residue-residue couplings in the Fyn SH2 domain, we employed a FoldX based sidechain sampling on the backbone structures of this SH2 domain as determined by NMR on the protein domain in isolation and bound to its phosphopeptide ligand (pdb identifiers 1AOU and 1AOT [38], see Methods). The NMR ensemble was determined using over 90% of the structurally non-redundant nuclear Overhauser effects (NOEs), so that the ensemble can be considered to possess adequate precision and accuracy [39-41]. In order to ensure reliable statistics, we collected over 551 thousand samples of sidechain configurations from approximately 275 million simulation steps over the 22 backbone models present in the NMR ensemble, producing the probabilities of finding the residues' sidechain in a particular conformation. From all these probabilities mutual information between all residue pairs was derived.

To capture how the information exchange within the SH2 domain changes due to ligand binding we require NMR structural data on both the bound and unbound state of the protein. Since there is no structural data on the unbound state of Fyn SH2 available publically, we assume here that the backbone flexibility for bound and unbound state can be derived from the ensemble stored in the 1AOU dataset by energy minimization (see Methods). This simplification has as result that large conformational changes in the backbone of the SH2 domain are not taken into account. Yet since domains do normally not experience large structural changes, this simplification may still provide viable results.

### Changes in mutual information link residues at long distances

Figure 2A shows for each residue couple the calculated change in mutual information upon phosphopeptide binding. As the figure shows, most residues do not experience a big change in mutual information (light blue regions in matrix. This fact is also visualized in Figure 2B, where we show the distribution of information change. Most changes in the information exchange between residue pairs falls in the interval [-0.5,0.5] bits. Since this set is almost not affected by binding, we will refer to this collection of residues as the silent group (for instance a serine at position 23, referred to as Ser23, near the end of the αA-helix in Figure 1B and 1D) and all values within this interval are considered to be noisy, meaning that a clear signal is difficult to obtain when the value of change falls in this range. Clearly, as a result of binding, all residues experience some (small) change. Yet, those residues that are more strongly affected are considered to be more relevant for understanding the information exchange inside the structure. As can be observed, much less residues experience strong effects and they correspond either to a (drastic) increase in coupling (green to red colors) or a (drastic) decrease in coupling (dark blue). These residues will be called the informative group. For instance HisβD4 at position 60 (residue in phosphotyrosine binding cavity, see Figure 1C) is one residue in the informative group whose coupling with all other residues increases significantly upon peptide binding. We also observe that, in case of the SH2 domain only a few residues seem to experience uncoupling as a result of phosphotyrosine binding: Tyr89 (see Figure 1C). Even though, little uncoupling is observed in Fyn SH2, this does not mean that significant uncoupling may not occur for other protein models, especially in the event of larger, binding-induced allosteric changes. The mutual information calculations introduced here can thus, in principle, capture very different mechanistic scenario's.

To visualise the mechanistic implications for our model system a clustering algorithm (see Methods) was applied on the mutual information matrix in Figure 2A and mapped on the structure of Fyn SH2. Figure 2C displays the residue cluster for which the mutual information increases most upon peptide binding (≥1.1 bits). Only those pairs of residue whose mutual dependence is above this threshold are accepted in the cluster, meaning that they form a complete graph where the weight of each link is above the threshold value. On the one hand, and as expected, these are composed of residues directly interacting with the phosphopeptide itself (ThrEF1, ThrEF2, HisβD4 and TyrβD5) but interestingly also consists of residues on the other side of the domain from which the SH2-SH3 and SH2-kinase linkers emerge (Pro27 and Pro103 according to PDB 1AOT). Even though previous studies of the Fyn SH2 domain [38] did not assign an important role to the conserved HisβD4, our analysis suggests this residue experiences a very strong change in mutual information upon ligand binding with all other residues in the structure (Figure 2B, yellow to red colors). This strong effect may be the result of the displacement of the helix αA in this particular SH2 domain, making the pTyr-binding cavity narrower when the peptide is bound [38]. Most affected seems to be residue ThrEF1.

Thus, our analysis reveals a strong coupling between the peptide binding site of Fyn SH2 and its SH3-SH2 and SH2-Kinase linkers. These first results show that the sampling approach discussed here provides a direct way to identify and quantify highly coupled groups of residues. Moreover, it confirms the notion that subtle changes in structural dynamics can effectively couple residues at distal locations in the structure. Note that the clustering does not show how this information exchange actually occurs, i.e. it does not provide a causal explanation. It provides an identification of the residues that may be involved with signaling. Yet, the idea is that when, by lowering the threshold, a coherent collection is obtained, possibly identifying a consecutive path that links the binding region to other parts of the domain structure.

### Closer inspection of the information exchange for some residues

**Figure 3:**
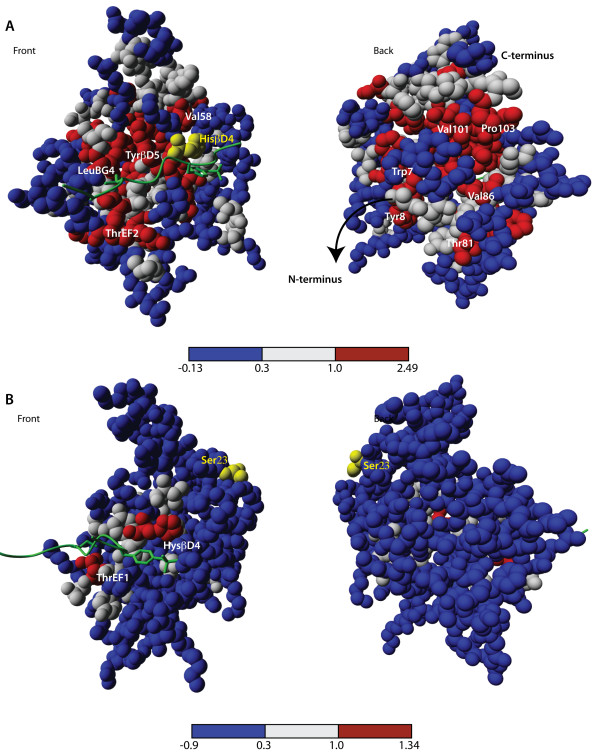
**Change in information exchange for HisβD4 and Ser23**. Both panels show a color coded visualization of the change in mutual information between a particular source residue and the all the other residues in the structure. Strongly coupled residues are colored red, weakly coupled residues are colored blue and intermediately coupled residues are colored white. **(A) **the results for HisβD4 and **(B) **the results for Ser23. All molecular graphics created with YASARA  and PovRay .

**Figure 4:**
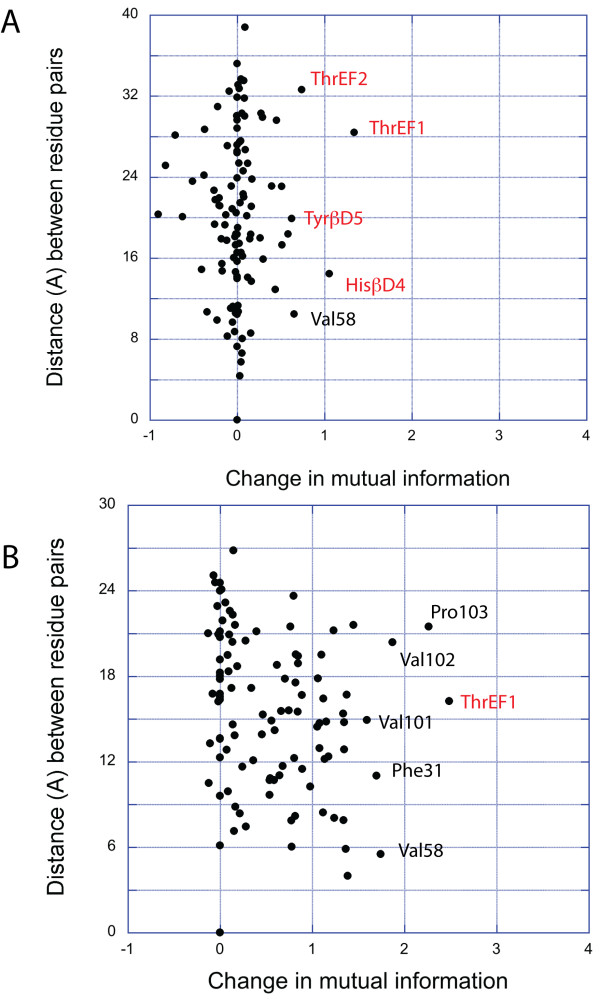
**Interaction distances of between residue sidechains**. Both pictures show the relation between change in mutual information and the distance between the centroids of the side chains of all the residues of the Fyn SH2 domain. Residues annotated in red correspond to residues in one of the binding cavities (see Figure 2C) **(A) **provides the results Ser23 and **(B) **provides the results for HisβD4.

To understand the key elements of information exchange in proteins we compared the change in mutual information of both silent and informative residues with the rest of the protein. We will here illustrate our analysis by two examples that recapitulate the key features of both informative as well as silent residues. Ser23 will serve as an instance of the collection of silent residues whereas HisβD4 represents a prominent member of the informative residues. Specifically, we mapped the change in mutual information of these residues with all the other residues of the SH2 domain onto the structure. Strong changes (ΔI > 1.0) are colored red, weak changes (ΔI < 0.3) blue and intermediate changes are white. In Figure 3A, we observe for HisβD4 that three classes of residues contribute significantly to its score. First, locally interacting residues directly influence each other (e.g. residue Val58). Second, the phosphorylated peptide connects all the relevant residues in the binding pocket, creating a channel by which conformational fluctuations can be transmitted (e.g. residue ThrEF1). As a consequence, these residues are mutually dependent on the state of HisβD4. Third, HisβD4 is also coupled to a number of residues at the other side of the protein. We see in Figure 3A (right) that the residues at the kinase linker (carboxyterminal end of the protein) that were previously detected by clustering are again present, i.e. long-range communication between the histidine and residues Val101, Pro103 and Val86 is clearly visible here. Moreover we see also a coupling with a number of residues related to the SH3-SH2-linker region (Trp at position 7 and Tyr at position 8 in the 1AOT structure), meaning that information is exchanged with both linker regions when switching between unbound and bound state. The results for Ser23 are strikingly different. In Figure 3B, we see that when the phosphorylated peptide becomes attached to the SH2 domain, only a few residues located in the peptide binding site exchange more information with this particular residue. Further, informative and silent residues do not only strongly differ in terms of the amount of residues with whom they are coupled but also in terms of the distances over which they communicate. Figure 4 shows that whereas Ser23 is conformationally isolated, HisβD4 has an extensive network of couplings with both proximate as well as distal residues.

### Contiguous communication pathway in Fyn SH2

As argued in previous sections, our information theoretical approach provides a method to quantify the change in exchange of mutual information between all pairs of residues of the Fyn SH2 domain as a consequence of ligand binding. Our analysis revealed that for the majority of residues the conformational coupling to the rest of the protein is not altered upon peptide binding and as a consequence they can be considered silent in terms of signal transduction. A small fraction of residues, however, experience a significant change in conformational coupling and are thus information-rich. Clustering only the most informative of these residues (ΔI ≥ 1.1 bits) revealed a strong coupling between the peptide binding site and the region harboring the linkers connecting the Fyn SH2 to the other domains of the Fyn kinase. It remains of course to be explained by which structural mechanism these distal sites become coupled. As argued earlier, the current framework can only identify and quantify the residues that are involved in the interaction. To identify the causal relationships a different analysis is required.

Several mechanisms for signal transduction have been described in the literature [30] and as such our method does not provide the means to unequivocally single out a given mechanism. However, clustering the informative residues and mapping these on the structure of the protein can provide substantial information. For instance the relative occurrence of coupling versus uncoupling pairs and their disposition on the topology of a protein domain could allow to distinguish pathway models [7,30] from allosteric models [42].

**Figure 5:**
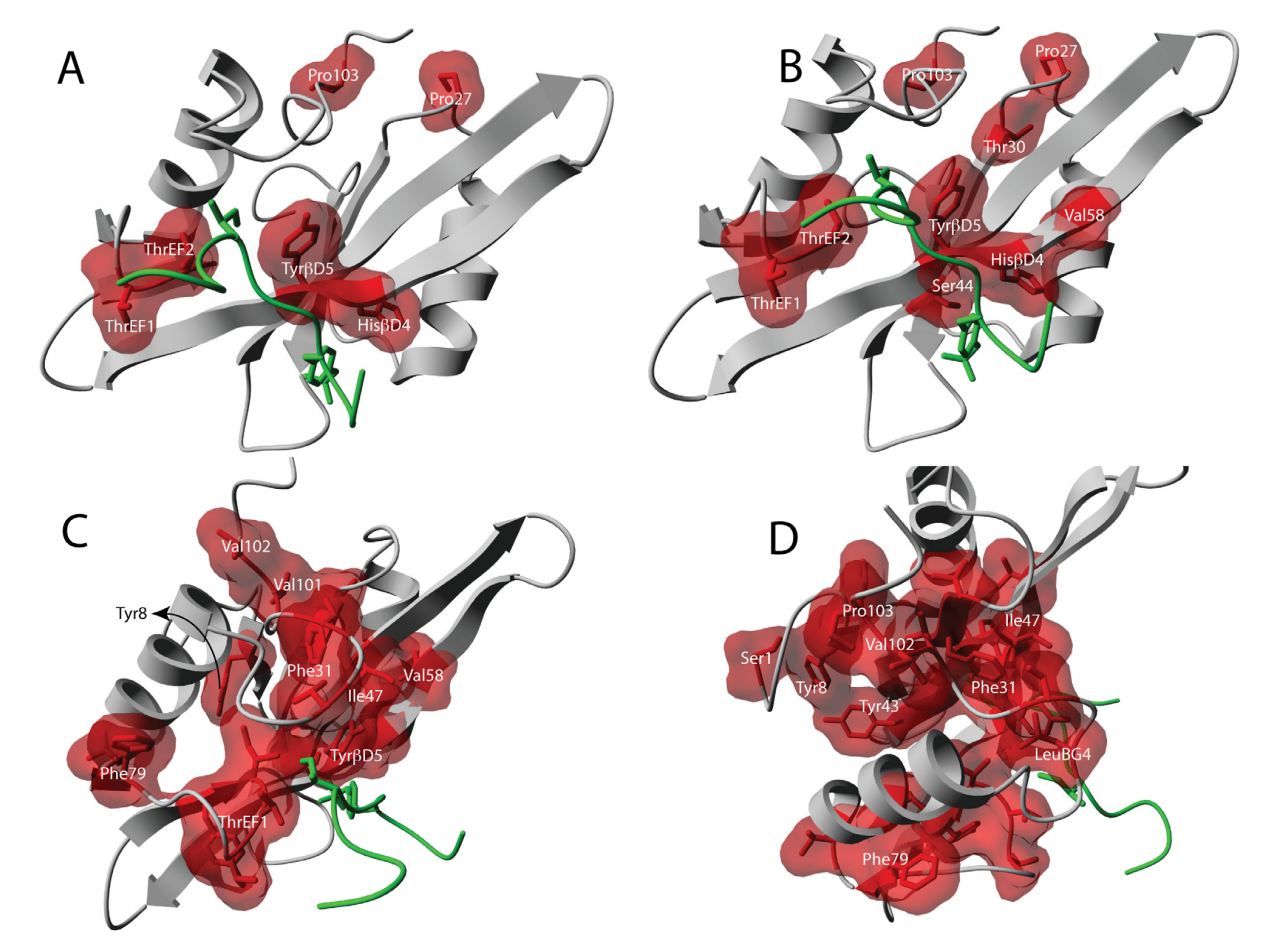
**Expanding the cluster of highly coupled residues**. As the cut-off for the clustering algorithm is decreased, more an more residues become involved, creating a pathway that connects the binding area with the linkers of the SH2 domain. Cutoffs for panel **A**, **B C **and **D **are respectively 1.1 bits, 0.9 bits, 0.7 bits and 0.5 bits. All molecular graphics created with YASARA  and PovRay .

As our model only displays increase in conformational coupling upon ligand binding, suggesting a pathway model, we here extract the group of residues that define the pathway through clustering (see Methods) of informative residues that have a mutual affinity higher than a particular noise level (0.5 bits). In Figure 5(A–D) we see how the pathway is formed by decreasing the clustering threshold from 1.1 to 0.5 bits: a contiguous dynamic pathway [30] emerges, involving the previously identified residues in the peptide binding site and the linker region.

As discussed above only a subset of residues of the SH2 domain is involved in signal transduction, whereas the other residues seem to be conformationally isolated from ligand binding. Importantly, signal transduction is achieved throughout the core of the SH2 domain and involves the main secondary structure elements of the structure. Hence, we show that the Fyn SH2 domain acts as indivisible information transmission unit propagating information from its binding site over the tertiary structures to the linker regions of the domain.

## Conclusion

Driven by the availability of genomics and proteomics data, biological research is increasingly focused on cellular networks rather than on individual proteins. Accordingly, a major effort in systems biology is devoted to understanding how topology shapes macromolecular networks into functional units performing information processing tasks such as logical gating, band-pass filtering or signal modulation. Biological networks are generally represented by graphs of interconnected protein nodes. In reality, however, these networks form macromolecular complexes whose assembly is determined by intermolecular atomic interactions. As shown by several experimental and theoretical studies, ligand binding may induce changes in the structural dynamics of proteins. These perturbations propagate cooperatively throughout the protein, coupling distal locations and thereby effectively transferring information. Hence, proteins domains are the elementary units of information processing in cellular organisms. As such proteins can themselves be represented as networks of interconnected residues exchanging information. Protein complexes can by extension be modeled in a similar way. The method introduced here allows to quantitatively translate protein structural dynamics in terms of Shannon's information theory. Moreover, mutual information permits to determine how much information is exchanged between each pair of residues within a protein structure. Transposing structural dynamics in terms of Shannon information has the advantage of providing a description level that matches the relevant functional features – i.e.biological information,- that we aim to extract from signal transduction pathways. Moreover, the analysis does not make any assumptions on the information transmission model. It strictly focuses on identifying the residues whose coupling increases or decreases as a consequence of peptide binding. Further, the method is applicable in identical manner to residue networks at atomic scale as well as to macroscopic descriptions of cellular networks.

As explained in Results, the mutual information exchanged between residues in protein residue networks is simply a statistical thermodynamics description of the conformational coupling between these residues. By discretising conformational space at the residue level, we easily find the average Shannon information for each residue by calculating the average entropy of the conformational probability distribution for each residue. The mutual information exchanged between two residues is the difference of the Shannon entropy from one residue and the conditional Shannon entropy of that residue given knowledge of the probability distribution of the other residue. In other words, it represents the amount of coupling between two protein residues. Once the mutual information is calculated for each pair of residues, clustering can be applied to determine the structural mechanism by which information is transferred between functional sites. In effect we hereby consider a protein as an instance of a noisy coding channel linking a probability distribution of input signals at one binding site with a probability distribution of output signals at another binding site. For the Fyn SH2 domain, we find that information between functional sites is conveyed by a contiguous pathway of residues that cross the hydrophobic core to connect both sites. In particular our results suggest a clear information flux between both binding sites and residues near the linkers of the domain towards the other domains of the Fyn kinase. As a result it is likely that removal of the phosphopeptide from the SH2 binding site uncouples the SH2 domain from both the SH3 domain as well as from the kinase domain, thereby facilitating the activation of the Fyn kinase as already suggested in the literature [25]. Importantly, communication between functional sites does not necessarily have to occur via a pathway mechanism. Other modes of signal transmission may equally be applicable in which the uncoupling of certain residues upon ligand binding plays a more important role (see for instance [30]). For instance, allosteric changes could result in a shift of couplings which could be identifiable by clusters of increased as well as decreased mutual information (e.g cracking [42]).

Using experimental methods such as NMR in combination with protein engineering approaches together with our method for information quantification should allow to test and explicit signal transduction mechanisms for a variety of model systems, which would be highly valuable especially for modular domains such as SH2, SH3 or PDZ domains. Especially for the latter domain a number of experimental studies have already shown the presence of long-range communication [43-45] Finally, it could contribute to understand how disease mutants and SNPs modulate the efficiency of information transfer by signaling proteins and whether for certain mutations this correlates with disease penetrance

## Methods

### PDB structures used in this analysis

In this work an NMR ensemble and average minimized structure of the Fyn SH2 domain was used [38]. Like the other members of the family, Src [46] and Hck [47], this domain binds, with high affinity, with ligands that have C-terminal to the phosphotyrosine two glutamates (+1 and +2) and an isoleusine (+3). Different from other SH2 structures is the critical role of GlyBG3 in the creation of the binding pocket. The actual NMR structure was constructed through high-resolution NMR spectroscopy from p59^fyn^, containing residues 143 to 248, in complex with the ligand EPQpYEEIPIYL (corresponding to residues 321 to 331 of the hamster polyomavirus middle T antigen). The structures of the ensemble (pdb identifier 1AOU) were selected on the basis of three criteria: They have the lowest energy score, no NOE violations above a 0.3 Angstrom threshold and no dihedral violations above 5 degrees. The structure in 1AOT is the energy minimized average structure for this domain. Sequence alignment shows that the structure also includes parts of the SH3 (residues 1 to 6) and Kinase (residues 90–106) linkers.

The energetic properties of all structures in the NMR ensemble were determined using the FoldX forcefield [36]. We observed high Vanderwaals repulsion energies, indicating clashes between the sidechains in the structure. To alleviate this problem all structures were energy minimized using the Yasara environment with the Yamber2 force field [48]. Resulting structures were reevaluated with FoldX showing highly improved overall energy scores (due to among others low Vanderwaals repulsion energies) and improved sidechain entropy.

**Figure 6:**
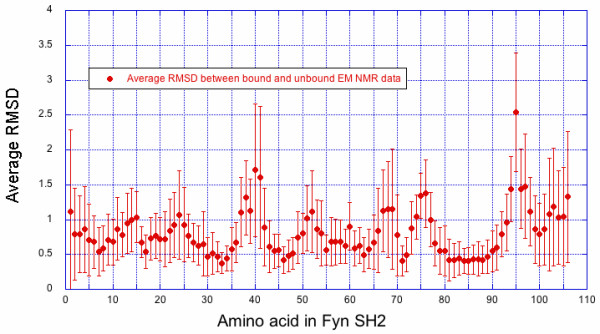
Average RMSD difference between bound and unbound structures of the Fyn SH2.

Since no data is publically available on the unbound domain structure of Fyn SH2, we derived an ensemble from the existing 1AOU data by first removing the peptide and then energy minimizing the structures using the Yamber2 force field. Figure 6, shows the average RMSD per residue between the different unbound and bound structures of both ensembles. Structural variations are larger at the termini of and the loop regions in the structure. Using this data introduces a simplification, i.e. we assume no large structural changes between bound and unbound states.

### Calculating mutual information

Mutual information expresses the amount of information that the output conveys about the input (and vice versa). It is formally expressed in terms of entropy:

(1) $$\begin{array}{l} I(X;Y)=I(Y;X)=H(X)+H(Y)-H(X,Y)\\ \begin{array}{ccc} H(X)=-\sum_{x\in X}p(x)\log p(x) & \text{and} & H(X,Y)=-\sum_{x,y\in X,Y}p(x,y)\log p(x,y) \end{array} \end{array}$$

All entropy values can be easily derived from the probabilities related to the conformational states of the residues. For instance, if X corresponds to a particular residue, then P(X = x) correspond to the probability if finding the residue's sidechain in conformation x and P(X = x, Y = y) corresponds to the probability of finding residues X and Y in conformation x and y, respectively. This probabilistic information is gathered by the Monte Carlo sampling discussed later.

Since the number of conformational states depends on the size of the amino acid sidechain, the actual mutual information values can differ. This kind of bias towards residues with large absolute values, which are predominantly the larger, partially solvent exposed sidechains, needs to be removed. To normalize the results, a measure called redundancy is used. Redundancy is calculated as $R=\frac{I(X;Y)}{H(X)+H(Y)}$, which has zero as its minimum and $R_{\max}=\frac{\min\left\{H(X),H(Y)\right\}}{H(X)+H(Y)}$ as maximum. Hence, to normalize all I(X;Y) to the interval [0,1], we first determine R and R_max _for every residue pair and then rescale this value to the interval taking the redundancy and its maximum value. This produces a normalized mutual information $I_{norm}(X;Y)=\frac{I(X,Y)}{\min\left\{H(X),H(Y)\right\}}$ that will be used to calculate the change in mutual information in our analysis.

### Dataset of backbone-dependent sidechain dihedral angles

We derived a statistical rotamer database based on conditional statistics of dihedral angles. All statistics were derived from the WHAT IF dataset [49] of single chains presenting unique structures, with a R-factor less than 0.2 and a resolution less than 1.9 Å (1344 structures at the end of 2003). We extracted out of this dataset all amino acids and their dihedral angles. Each dihedral angle was divided in 10° bins, and we derived the following probabilities: P(Chi_i_), P(Chi_i _| Chi_i-1_) and P(Chi_i_| Chi_i-1_, Chi_i-2_), except for Chi_1 _(P(Chi_1_) and P(Chi_1_|Phi, Psi)).

A set of n random rotamers can be asked from the database and will be derived from the probability distribution previously calculated. The first dihedral angle, Chi_1_, will be derived first depending on the backbone conformation of the actual residue to mutate (Phi, Psi angles). If the position in the structure is on a poorly populated area of the Ramachandran plot (according to the WHAT IF dataset), we use statistics derived from neighbor bins (+/- 20° on each dihedral angles, keeping the most populated bin) and a small fraction (10%) of the sidechains will also be generated using independent probabilities (P(Chi_1_)). Chi_2 _angles are generated according to the single conditional probability distribution P(Chi_2_|Chi_1_) based on the previously determined dihedral angle. For longer sidechains, i.e. for Chi_3 _and Chi_4 _angles, the random dihedrals are generated according to the probability distributions P(Chi_i _| Chi_i-1_, Chi_i-2_).

In the context of the work presented here, the aim is not to create a rotamer library per se, but more importantly to represent the conformational space of a residue X as a discrete alphabet or state space. The approach used here allows enumeration of states with greater resolution than classical rotamer libraries. A similar set of higher resolution sidechain conformers were used by Honig and co-workers with great success for sidechain reconstruction [50].

### Monte Carlo sampling of sidechain conformations

Before starting the sampling process, the alphabet of possible sidechain conformations for each residue is determined using the method discussed in the previous section. The process iterates over all backbones in the NMR data, checks whether the conformation in the data is present and adds it if it does not yet exist, meaning it has a 10 degrees difference with the conformations already in the alphabet. Once this is determined the same alphabet is used for the sampling of every backbone in the NMR ensemble.

One run off the sampling process, which uses the FoldX force field to determine the energy of each state and a Monte Carlo algorithm to perform the conformational sampling, is given one backbone from the NMR data, commences with an energy minimization phase, to ensure that the sampling starts from a state in the energy well and then start collecting data of the residue states. In order to collect many data points this process is run many times in parallel for every backbone separately.

Details and concept of the FoldX force field are discussed at length by Guerois et al. [51]. The most important modifications in the 2.6 version are listed in Schymkowitz et al. [36,37]. Metropolis Monte Carlo sampling of sidechain conformation was performed at 298 K by sampling sidechain conformations randomly according to the probability distribution of the backbone-dependent side-chain dihedral angles. The Metropolis criterion states that a certain conformational change is accepted with a probability p that depends on the free energy change ΔG associated with the conformational change as given by the following formula:

*P *= min {1, exp(-Δ*G*/*RT*)}

Note that FoldX is used to score the complete conformation including the packing interactions.

### Relative change in mutual information

In the core of the protein changes in conformational dynamics are smaller in absolute value since the alphabet for smaller hydrophobic residues is limited and the conformational restrictions from the neighbouring residues is much more pronounced. Nevertheless, small absolute changes in such positions may still represent large relative changes. Thus, a relative scoring of the change in information exchange will separate meaningful signals from meaningless signals in a better way. Using a logarithm of the ratio of bound mutual information versus unbound mutual information is hence a reasonable way to represent this relative change in mutual information.

### Clustering the highly coupled residues

The matrix of change in mutual information between all residue pairs is clustered using the CAST algorithm [52]. This algorithm was originally constructed to cluster genes with coupled expression profiles. The algorithm generates a number of clusters of highly coupled elements based on an affinity measure. Affinity is defined here by $a(x)=\sum_{y\in R}I(x,y)$, where R is the set of residues belonging already to the cluster and I(x, y) the mutual information change between residue x and residue y. The acceptance criterion (or affinity threshold) is the parameter t. Residues with high affinity (*a*(*x*) ≥ *t*|*R*|) are added to the cluster and low affinity residues are removed. The algorithm alternates between both steps until the cluster R stabilizes. The benefit of this approach to other algorithms like hierarchical clustering is that it can undo bad additions of residues to a particular cluster.

## Authors' contributions

TL contributed in the conception and development of the principles of this work, developed the computational framework and carried out the computational analysis. JFB, FS, LS, JS and FR conceived the principles of the work and assisted in the development of the computational framework. All authors drafted, edited and approved the manuscript.
